# Synthesis of Pyrrolo[1,2-*a*]pyrimidine Enantiomers via Domino Ring-Closure followed by Retro Diels-Alder Protocol

**DOI:** 10.3390/molecules22040613

**Published:** 2017-04-13

**Authors:** Beáta Fekete, Márta Palkó, Matti Haukka, Ferenc Fülöp

**Affiliations:** 1 Institute of Pharmaceutical Chemistry, University of Szeged, Eötvös utca 6, Szeged H-6720, Hungary; fekete.beata@pharm.u-szeged.hu (B.F.); palko@pharm.u-szeged.hu (M.P.); 2 Department of Chemistry, University of Jyväskylä, FIN-40014 Turku, Finland; matti.o.haukka@jyu.fi; 3 MTA-SZTE Stereochemistry Research Group, Hungarian Academy of Sciences, Eötvös utca 6, Szeged H-6720, Hungary

**Keywords:** domino reactions, hydroxamic acid, microwave chemistry, *N*-heterocycles, retro Diels-Alder reaction

## Abstract

From 2-aminonorbornene hydroxamic acids, a simple and efficient method for the preparation of pyrrolo[1,2-*a*]pyrimidine enantiomers is reported. The synthesis is based on domino ring-closure followed by microwave-induced retro Diels-Alder (RDA) protocols, where the chirality of the desired products is transferred from norbornene derivatives. The stereochemistry of the synthesized compounds was proven by X-ray crystallography. The absolute configuration of the product is determined by the configuration of the starting amino hydroxamic acid.

## 1. Introduction

The first hydroxamic acid (oxalohydroxamic acid [1]) was discovered by Lossen as early as 1869. However, hydroxamic acids attracted further attention at the beginning of the 1980s because of their bioactivity [2]. Their pharmacological properties are related to their ability to scavenge metal ions [3]. In addition, they are able to generate nitric oxide [3,4,5] in living systems. In this way, they can act as antimicrobial [6,7,8,9], antitumour [6,10], antihypertensive [11], anti-inflammatory [6,12], and neuroleptic agents, among others [3,13].

According to the literature data, aromatic hydroxamic acids are useful compounds [14,15,16,17,18,19,20,21,22,23]. However, only limited information is available about their reactions [24,25] and, in particular, about the properties of alicyclic derivatives [26,27].

Though the hydroxamic acid moiety is an important key pharmacophore in most cases, it is usually built up just in the last step of the synthesis [28,29,30]. Previously, we have examined the reactivity and stereoselectivity of the domino reaction of 2-aminonorbornene hydroxamic acids with 2-formylbenzoic acid and 2,3-dimethoxy-6-formylbenzoic acid to form new isoindolo[2,1-*a*]quinazolines and pyrimido[2,1-*a*]isoindoles [31].

Focusing on the biological potential of fused quinazolinones, and continuing our work on the synthesis of novel *N*-heterocycles, herein, we report the synthesis of a new series of pyrrolo[1,2-*a*]quinazolinones and pyrrolo[1,2-*a*]pyrimidines starting from hydroxamic acids. Pyrroloquinazolines and pyrrolopyrimidines are important heterocyclic ring systems that occur as a core structure in a variety of naturally occurring alkaloids and synthetic compounds. Pyrrolo[1,2-*a*]quinazolines are tricyclic compounds with great potential and combine the quinazoline substructure, a privileged structure in medicinal chemistry, with a pyrrole [32]. Their synthetic methods are rather scarce but the past five years have brought efficient new synthetic strategies which could lead to an increased interest in pyrrolo[1,2-*a*]quinazolines in the near future, mainly for their potential applications in medicinal chemistry [33,34,35,36,37,38].

Pyrrolopyrimidines display a broad applicability in medicinal chemistry exhibiting antimicrobial [39,40,41,42,43], antitumour [44,45,46,47,48,49,50,51,52,53,54,55,56,57,58], antiasthmatic [59], antihypertensive [60], and anti-inflammatory [61] activities. Several method have been developed for synthesizing pyrrolopyrimidines in the last few years [62,63,64,65,66,67,68,69,70].

We recently reported an efficient and convenient procedure for the preparation of pyrrolo[1,2-*a*]pyrimidines starting from 2-aminonorbornene hydroxamic acids [31]. The synthesis was based on the domino ring-closure reaction of hydroxamic acids with oxocarboxylic acids, followed by RDA reaction of the tetracyclic intermediates.

The present target derivatives were planned to be prepared by a two-step strategy: first, the domino ring-closure reaction of *diendo-* and *diexo-2-*aminonorbornene hydroxamic acid with levulinic acid and α-ketoglutaric acid was carried out, followed by the second step involving the RDA reaction of the formed tetracycles by the loss of cyclopentadiene.

Domino reactions with hydroxamic acids are not well-documented in the literature. There are examples for the main simple ring-closures with cyclic anhydrides [19], aldehydes [71], sodium nitrite [20], carbon disulfide [72], formic acid, or acetyl chloride [17].

Our present aim was (i) to examine the domino ring-closure reaction of *diendo*- and *diexo*-aminonorbornene hydroxamic acids **1** and **2**, (ii) to develop the retro Diels-Alder reaction of the tetracyclic heterocycles formed, and to extend this methodology to obtain novel racemic and enantiomeric pyrrolo[1,2-*a*]pyrimidine derivatives.

## 2. Results and Discussion

Racemic *diendo*- and *diexo*-2-aminonorbornene hydroxamic acids (±)-**1** and (±)-**2** were prepared from the appropriate ester bases with aqueous hydroxylamine solution according to an earlier procedure [31]. The enantiomers of 2-aminonorbornene hydroxamic acid (+)-**1**, (–)-**1,** (+)-**2**, and (–)-**2** were prepared from racemic esters via diastereomeric salt formation with *O*,*O*’-di-*p*-toluoyl-tartaric acid (DPTTA) and *O*,*O*’-di-benzoyl-tartaric acid (DBTA) as previously described [31].

In the optimization experiments of the domino reactions of levulinic acid and α-ketoglutaric acid with racemic *diendo*-2-aminonorbornene hydroxamic acid (±)-**1** and *diexo*-2-aminonorbornene hydroxamic acid (±)-**2**, conventional heating or microwave irradiation was applied. The reaction time was significantly shorter in the microwave reactor: the best results were achieved in ethanol stirred at 100 °C for 1 h.

**Scheme 1:**
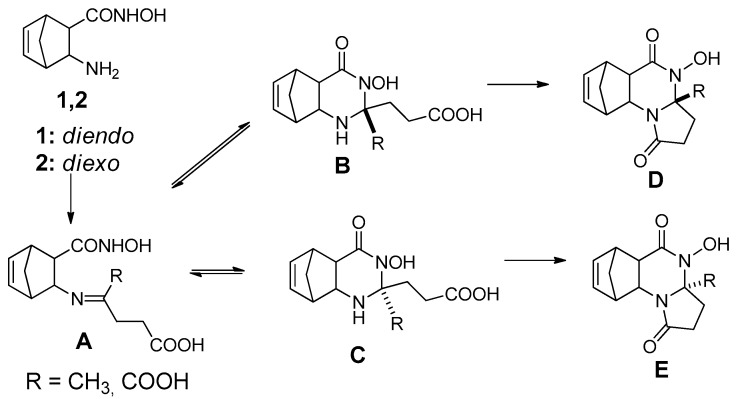
The domino reaction pathway.

In the domino reaction of **1** and **2** with levulinic acid and α-ketoglutaric acid, the first Schiff base **A** is produced, which undergoes a ring-closure reaction to produce the quinazoline epimers **B** and **C**, which are formed from **A** through a ring-chain tautomerism. The second ring-closure involves epimers **B** and **C** that yields compounds **D** and **E** (Scheme 1) [68].

**Scheme 2:**
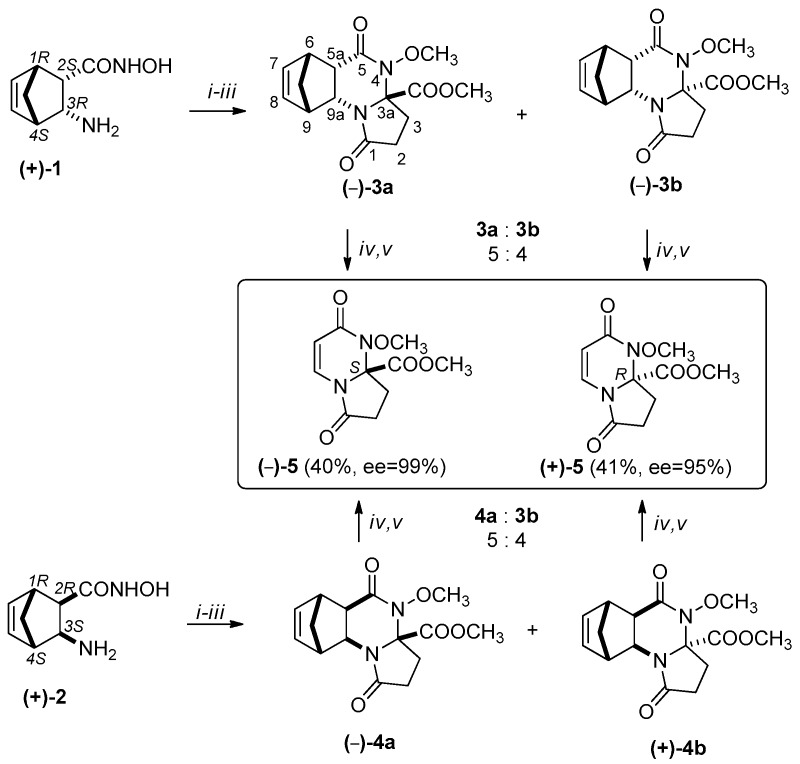
Synthesis of (+)-**5** and (–)-**5**: (i) *α*-ketoglutaric acid, 1 h, 100 °C, EtOH, Microwave irradiation (MW); (ii) MeOH, diazomethane/Et_2_O, Room temperature (r.t.) (iii) separation of major (–)-**3a**, (–)-**4a** and minor (–)-**3b**, (+)-**4b** epimers by column chromatography (epimeric ratio **3a**:**3b** =5:4, **4a**:**4b** = 5:4, eluent: EtOAc) (iv) MW, 20 min, 250 °C, 300 W (v) column chromatography (eluent: EtOAc).

The NMR spectra revealed the formation of two diastereomers of the methanopyrrolo[1,2-*a*]quinazolines **D** and **E** (Scheme 1). Unfortunately, we were not able to separate the diastereomers by column chromatography despite the use of a range of eluent combinations (for example: EtOAc, EtOAc/hexane = 1:1, EtOAc/MeOH = 9:1, CHCl_3_/MeOH = 9:1, or toluene/MeOH = 4:1). Fortunately, after derivatization with diazomethane, the diastereomers (±)-**3a**, (±)-**3b** and (±)-**4a**, (±)-**4b** could be easily separated by column chromatography eluted with EtOAc (Scheme 2).

**Figure 1:**
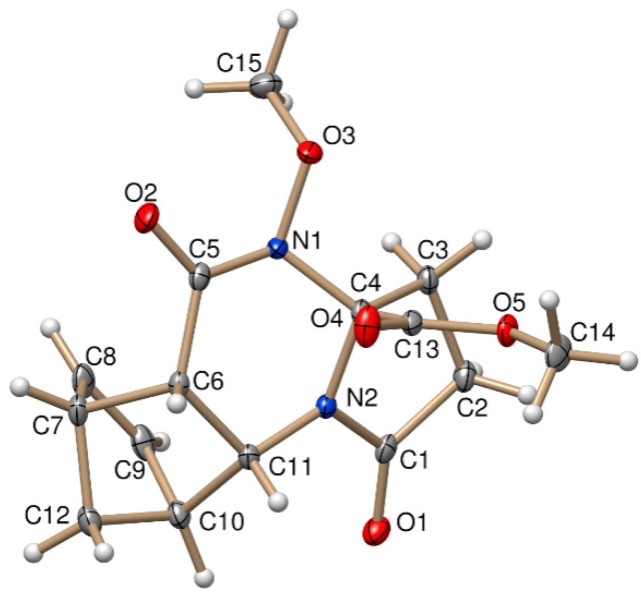
Oak Ridge Thermal Ellipsoid Plot (ORTEP) plot of the X-ray structure of (±)-**3a**.

**Figure 2:**
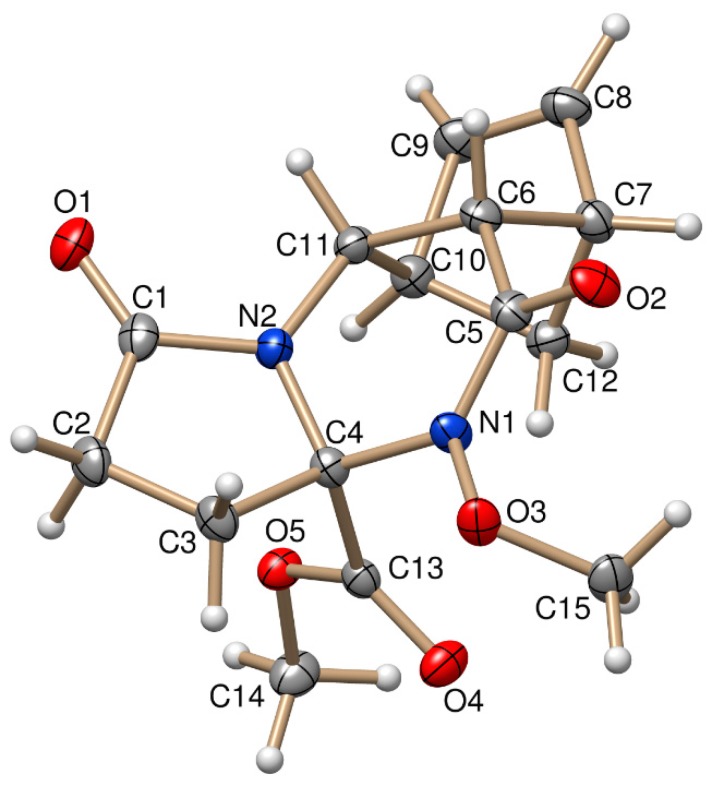
ORTEP plot of the X-ray structure of (±)-**4b**.

The stereochemistry of (±)-**3a** and (±)-**4b** was confirmed by X-ray diffraction analysis. The relative configuration of the COOCH_3_ group at C-3a and the bridgehead hydrogens of C-5a and C-9a have the same steric orientation in the (±)-**3a** and (±)-**4b** tetracyclic derivatives (Figure 1 and Figure 2).

In order to produce racemic pyrrolo[1,2-*a*]pyrimidine **5**, the retro Diels-Alder reaction of the methanopyrrolo[1,2-*a*]quinazoline derivatives was examined in the microwave reactor under varied conditions. The best results for the cycloreversion were achieved in 1,2-dichlorobenzene (DCB) at 250 °C (20 min).

The synthetic method described above was extended for the preparation of the enantiomerically pure substances, via the protocol described for the racemic substances. The domino ring-closure reaction of *diendo*-2-aminonorbornene hydroxamic acid enantiomer (+)-**1** resulted in (–)-**3a** and (–)-**3b**, while that with the *diexo*-2-aminonorbornene hydroxamic acid enantiomer (+)-**2** resulted in epimers (–)-**4a** and (+)-**4b**. After their separation, the RDA reactions of tetracycles could easily be achieved, resulting in pyrrolo[1,2-*a*]pyrimidine (+)-**5** and (–)-**5** (Scheme 2).

**Figure 3:**
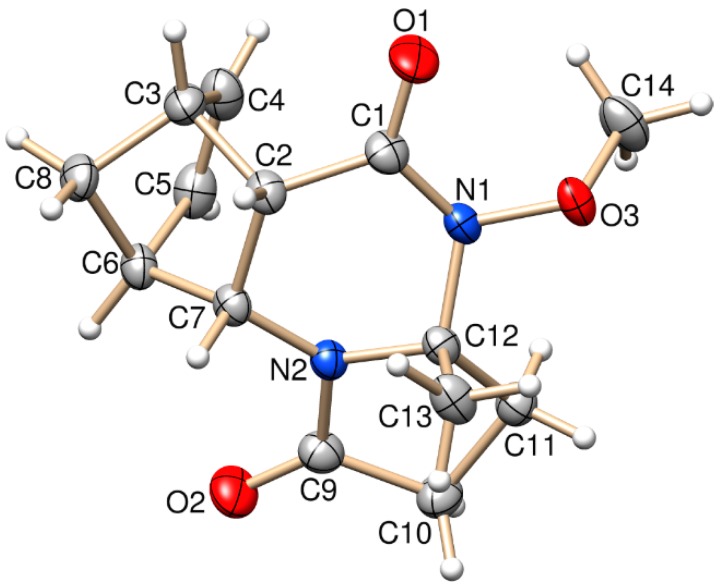
ORTEP plot of the X-ray structure of (±)-**6a**.

We attempted to use the same methodology to synthesize racemic **8**. In this case, however, the ring-closure reaction with levulinic acid resulted in only a small quantity of the minor diastereomers (±)-**6b** and (±)-**7b**, which could not be isolated. The stereochemistry of (±)-**6a** was confirmed by X-ray diffraction analysis. The relative configuration of the CH_3_ group at the C-3a asymmetric centre and the bridgehead hydrogens of C-5a and C-9a have the same steric orientation in (±)-**6a** (Figure 3).

**Scheme 3:**
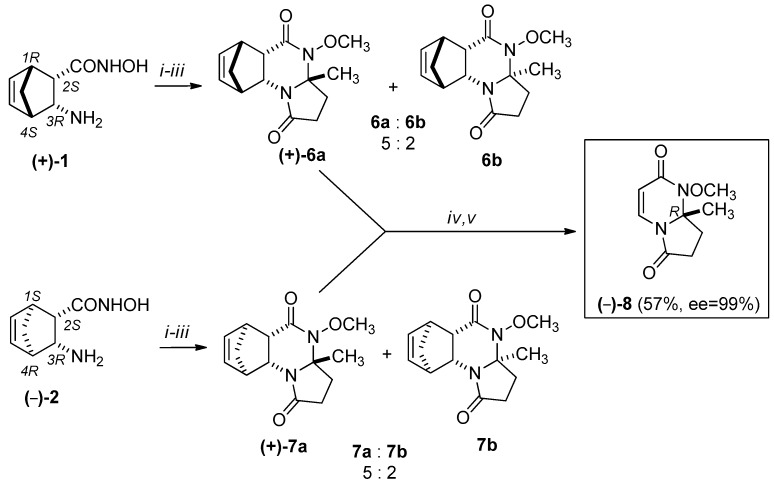
Synthesis of (–)-**8**: (i) levulinic acid, 1 h, 100 °C, EtOH, MW; (ii) MeOH, diazomethane/Et_2_O, r.t.; (iii) isolation of major ((+)-**6a**, (+)-**7a**) epimers by column chromatography (eluent: EtOAc:MeOH = 9:1) (iv) Retro Diels-Alder (RDA) reaction of major epimers: MW, 220 °C, 20 min, 300 W, (v) column chromatography (eluent: EtOAc:MeOH = 9:1).

**Scheme 4:**
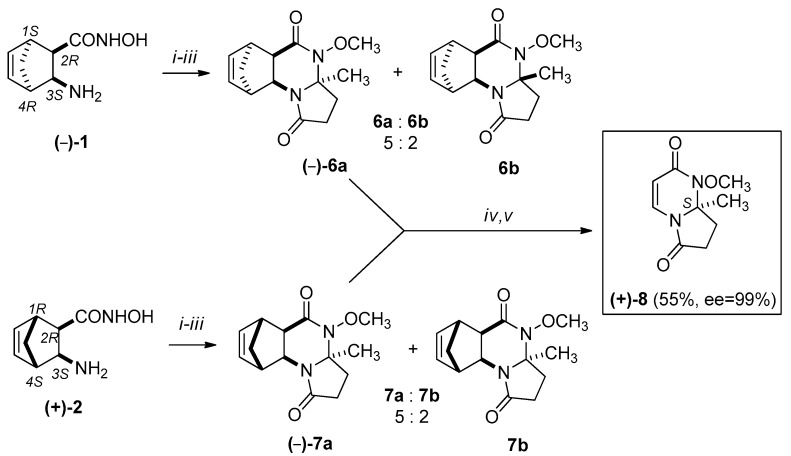
Synthesis of (+)-**8**: (i) levulinic acid, 1 h, 100 °C, EtOH, MW; (ii) MeOH, diazomethane/Et_2_O, r.t.; (iii) isolation of major (–)-**6a**, (–)-**7a**) epimers by column chromatography (eluent: EtOAc:MeOH = 9:1); (iv) RDA reaction of major epimers: MW, 220 °C, 20 min, 300 W, (v) column chromatography (eluent: EtOAc:MeOH =9:1).

We selected an economically better approach for the synthesis of (+)-**8** and (–)-**8**. Namely, we carried out the cycloreversions of the major methanopyrrolo[1,2-*a*]quinazoline derivatives (+)-**6a**, (–)-**6a**, (+)-**7a**, and (–)-**7a** and isolated the pyrrolo[1,2-*a*]pyrimidines (+)-**8** and (–)-**8** in 55–57% yields (see Scheme 3 and Scheme 4).

The absolute configuration of the newly built asymmetric centre of (+)-**7a** and (–)-**7a** was determined by chemical correlation: the absolute configurations of the starting 2-aminonorbornene hydroxamic acids, and the relative configuration of (±)-**6a** from the X-ray diffraction analysis was known. When (+)-**7a** was heated under the RDA reaction conditions, (–)-**8** was gained. In addition, the RDA product of (+)-**6a** also afforded (–)-**8**. In contrast, when (–)-**7a** and (–)-**6a** were treated under the RDA reaction conditions, (+)-**8** was formed. The NMR and HPLC data (retention times: (–)-**8**: 41.91 min, opposite enantiomer (+)-**8**: 34.19 min) and comparison of the optical rotations revealed that the final compound was identical to that of (*R*)-1-methoxy-8a-methyl-1,7,8,8a-tetrahydropyrrolo[1,2-*a*]pyrimidine-2,6-dione (–)-**8**. Since this asymmetric centre was not affected during the RDA reaction, these results allow for the assumption that the absolute configuration of position 3a of (+)-**7a** is *R*, and that of (–)-**7a** is *S*.

## 3. Materials and Methods

### 3.1. General Methods

^1^H-NMR spectra were recorded at 400.13 MHz or 600.20 MHz and the ^13^C-NMR spectra were recorded at 100.62 MHz or 150.92 MHz in CDCl_3_ at ambient temperature, with a Bruker AM 400 or Bruker AV 600 spectrometer (Bruker Biospin, Karlsruhe, Germany). Chemical shifts are given in δ (ppm) relative to TMS as the internal standard. Microwave-promoted reactions were performed in sealed reaction vials (10 mL) in a microwave (CEM, Discover SP) cavity (CEM Corporation, Matthwes, NC, USA). Optical rotations were measured with a Perkin-Elmer 341 polarimeter (Perkin Elmer, Shelton, CT, USA). Mass spectra were recorded with a Micromass Q-TOF Premier mass spectrometer (Waters Corporation, Milford, MA, USA). Melting points were measured with a Hinotek-X4 micro melting point apparatus (Hinotek, Ningbo, China) and are uncorrected. Racemic 2-aminonorbornene hydroxamic acids (±)-**1** and (±)-**2** and enantiomeric 2-aminonorbornene hydroxamic acids (+)-**1**, (–)-**1**, (+)-**2**, and (–)-**2** were prepared by a literature method [31]. The *ee* values of (+)-**1**, (–)-**1**, (+)-**2**, and (–)-**2** were determined by HPLC by a literature method [73]. CCDC-1508562-1508564 contains the supplementary crystallographic data for this paper. These data can be obtained free of charge from The Cambridge Crystallographic Data Centre via www.ccdc.cam.ac.uk/data_request/cif.

The *ee* values of (–)-**3a**, (–)-**3b**, (–)-**4a**, and (+)-**4b** were determined by HPLC using a Chiralcel-OD-H column (Daicel corporation, Tokyo, Japan). The analytical conditions were as follows: eluent: a mixture of hexane and EtOH (75:25) with 0.1% diethylamine, flow rate: 0.4 mL∙min^−1^, detection at 220 nm retention times: (−)-**3a**: 18.82 min (antipode: 22.16 min), (–)-**3b**: 19.84 min (antipode: 24.23 min), (+)-**4a**: 20.74 min (antipode: 17.48 min), (–)-**4b**: 24.22 min (antipode: 20.97 min). (+)-**6a**, (–)-**6a**, (+)-**7a**, and (–)-**7a** were determined using a Phenomenex-IA column (Phenomenex, Torrance, CA, USA) eluted by a mixture of hexane and IPA (70:30), flow rate: 0.4 mL∙min^−1^, detection at 220 nm, retention times: (+)-**6a**: 32.94 min, (–)-**6a**: 39.46 min, (+)-**7a**: 31.39 min, (–)-**7a**: 35.39 min). The *ee* values of the final products of (+)-**5**, (–)-**5**, (+)-**8**, and (–)-**8** were determined by HPLC using a Phenomenex-IA column. The analytical conditions were as follows: eluent: a mixture of hexane and IPA (70:30), flow rate: 0.4 mL∙min^−1^, detection at 220 nm, retention times: (+)-**5**: 47.97 min, (–)-**5**: 57.79 min, (+)-**8**: 34.19 min, (–)-**8**: 41.91 min.

^1^H-NMR and ^13^C-NMR spectra of compounds (–)-**3a,** (–)-**3b,** (–)-**4a,** (+)-**4b,** (+)-**5** (+)-**6a,** (+)-**7a** and (+)-**8**, HPLC chromatogram of compounds (±)-**5,** (–)-**5,** (+)-**5** (±)-**8** (–)-**8,** (+)-**8**, and table for X-ray crystallography data for (±)-**3a**, (±)-**4a** and (±)-**6a** are available in the Supplementary materials.

### 3.2. Synthesis of New Compounds

#### 3.2.1. Synthesis of Methanopyrrolo[1,2-*a*]quinazoline Derivatives (–)-**3a**, (–)-**3b**, (–)-**4a**, and (+)-**4b**

A mixture of 2-aminonorbornene hydroxamic acid (+)-**1** or (+)-**2** (336 mg, 2 mmol), and α-ketoglutaric acid (292 mg, 2 mmol) was dissolved in 6 mL EtOH in a 10 mL pressurized reaction vial and the solution was stirred at 100 °C for 1 h at max 300 W microwave irradiation. Then the reaction mixture was evaporated to dryness and the crude product was crystallized from Et_2_O. The crystals isolated were dissolved in MeOH and a diazomethane/Et_2_O mixture was added dropwise to the solution at ambient temperature and was stirred for 30 min. The reaction was followed by TLC. (Diazomethane is a very harmful and hazardous reagent and must be handled with caution! This reaction should be performed in a well-ventilated hood!) The solvent was then removed by evaporation and the residue was dissolved in 2 mL EtOAc and transferred to a silica gel column (Merck, Darmstadt, Germany) and eluted with EtOAc. The product was crystallized from *i*Pr_2_O to produce white crystals.

*Methyl (3aS,5aS,6R,9S,9aR)-4-methoxy-1,5-dioxo-1,2,3,3a,4,5,5a,6,9a-decahydro-6,9-methanopyrrolo[1,2-a]quinazoline-3a-carboxylate [(–)-**3a**)]:* White crystals (39% yield), m.p. 125–128 **°**C**,** ${\left[\alpha \right]}_{D}^{20\;}$ = –92.5 (*c* = 0.33, EtOH), *ee* 87%, ^1^H-NMR (400 MHz, CDCl_3_, 30 °C): 1.36–1.38 (m, 1H, 11-H), 1.58–1.60 (m, 1H, 11-H), 2.13–2.21 (m, 1H, CH_2_), 2.45–2.51 (m, 1H, CH_2_), 2.58–2.74 (m, 2H, CH_2_), 3.09–3.12 (m, 1H, 5a-H), 3.43 (m, 1H, 9-H), 3.82 (s, 3H, COOCH_3_), 3.85 (s, 3H, OCH_3_), 3.93–3.95 (m, 1H, 9a-H), 4.06 (m, 1H, 6-H), 6.07–6.09 (m, 1H, 8-H), 6.20–6.22 (m, 1H, 7-H), ^13^C-NMR (100 MHz, CDCl_3_, 30 °C): δ *=* 28.1, 30.1, 41.1, 46.6, 46.9, 46.9, 47.3, 54.0, 54.6, 65.3, 82.3, 135.7, 136.1, 170.0, 172.3, 173.9, HRMS calcd. for [M + H^+^] *m*/*z* = 307.1294, measured: *m*/*z* = 307.1288.

*Methyl (3aR,5aS,6R,9S,9aR)-4-methoxy-1,5-dioxo-1,2,3,3a,4,5a,6,9a-decahydro-6,9-methanopyrrolo[1,2-a]quinazoline-3a-carboxylate [(–)-**3b**)]:* White crystals (44% yield), m.p. 170–172 °C, ${\left[\alpha \right]}_{D}^{20}$ = −3.8 (*c* = 0.30, EtOH), *ee* 97%, ^1^H-NMR (600 MHz, CDCl_3_, 30 °C): 1.44–1.45 (m, 1H, 11-H), 1.56–1.63 (m, 1H, 11-H), 2.24–2.30 (m, 1H, CH_2_), 2.46–2.50 (m, 2H, CH_2_), 2.79–2.85 (m, 1H, CH_2_), 3.25–3.28 (m, 2H, 5a-H, 9-H), 3.42 (m, 1H, 6-H), 3.78 (s, 3H, COOCH_3_), 3.89 (s, 3H, OCH_3_), 4.68–4.69 (m, 1H, 9a-H), 5.73–5.75 (m, 1H, 8-H), 6.19–6.20 (m, 1H, 7-H), ^13^C-NMR (100 MHz, CDCl_3_, 30 °C): δ *=* 29.2, 32.1, 45.1, 46.3, 47.2, 48.9, 52.9, 53.1, 64.3, 81.6, 134.2, 138.6, 168.4, 170.3, 174.9, HRMS calcd. for [M + H^+^] *m*/*z* = 307.1294, found *m*/*z* = 307.1288.

*Methyl (3aS,5aR,6R,9S,9aS)-4-methoxy-1,5-dioxo-1,2,3,3a,4,5a,6,9a-decahydro-6,9-methanopyrrolo[1,2-a]quinazoline-3a-carboxylate [(–)-**4a**)]:* White crystals, (44% yield), m.p. 125–128 °C**,** ${\left[\alpha \right]}_{D}^{20}$ = −64 (*c* = 0.30, EtOH), *ee* 97% ^1^H-NMR (600 MHz, CDCl_3_, 30 °C): 1.39–1.40 (m, 1H, 11-H), 1.49–1.50 (m, 1H, 11-H), 2.40–2.56 (m, 3H, CH_2_), 2.65–2.81 (m, 3H, CH_2_, 5a-H, 9-H), 3.34 (m, 1H, 6-H), 3.84 (s, 3H, COOCH_3_), 4.02 (s, 3H, OCH_3_), 4.15 (m, 1H, 9a-H), 6.23–6.25 (m, 1H, 7-H), 6.31–6.33 (m, 1H, 8-H), ^13^C-NMR (150 MHz, CDCl_3_, 30 °C): δ *=* 29.1, 30.9, 44.4, 44.8, 46.9, 47.5, 51.9, 53.4, 64.0, 81.6, 137.6, 138.4, 167.7, 170.9, 173.9, HRMS calcd. for [M + H^+^] *m*/*z* = 307.1294, found *m*/*z* = 307.1288.

*Methyl (3aR,5aR,6R,9S,9aS)-4-methoxy-1,5-dioxo-1,2,3,3a,4,5a,6,9a-decahydro-6,9-methanopyrrolo[1,2-a]quinazoline-3a-carboxylate [(+)-**4b**]:* White crystals, (39% yield), m.p. 170–172 °C**,** ${\left[\alpha \right]}_{D}^{20}$= + 6.5 (*c* = 0.33, EtOH), *ee* 81%, ^1^H-NMR (600 MHz, CDCl_3_, 30 °C): 1.51–1.52 (m, 2H, 11-H), 2.25–2.30 (m, 1H, CH_2_), 2.58–2.82 (m, 4H, CH_2_, 5a-H), 3.35 (m, 1H, 9-H), 3.39–3.40 (m, 1H, 6-H), 3.81 (s, 3H, COOCH_3_), 3.94 (s, 3H, OCH_3_), 3.99 (m, 1H, 9a-H), 6.17–6.19 (m, 1H, 7-H), 6.33–6.34 (m, 1H, 8-H), ^13^C-NMR (150 MHz, CDCl_3_, 30 °C): δ *=* 27.9, 30.1, 43.7, 44.6, 46.6, 47.7, 53.7, 54.3, 65.0, 82.0, 135.7, 138.6, 169.4, 173.2, 174.1, HRMS calcd. for [M + H^+^] *m*/*z* = 307.1294, found *m*/*z* = 307.1288.

#### 3.2.2. Synthesis of Pyrrolo[1,2-*a*]pyrimidines (+)-**5** and (–)-**5**

Tetracyclic compound (–)-**3a**, (–)-**4a**, or (–)-**3b** and (+)-**4b** (50 mg, 0.16 mmol,) was dissolved in 2 mL DCB in a 10 mL sealed reaction vial. The solution was stirred at 250 °C for 20 min at max 300 W microwave irradiation. Then the solvent was evaporated, the residue was dissolved in 2 mL EtOAc/MeOH = 9:1, and was purified by column chromatography on silica gel eluted by EtOAc/MeOH = 9:1. The product was crystallized from *i*Pr_2_O to produce white crystals.

*Methyl (R)-1-methoxy-2,6-dioxo-1,2,6,7,8,8a-hexahydropyrrolo[1,2-a]pyrimidine-8a-carboxylate [(+)-**5**]:* White crystals, (41% yield), m.p. 156–158 °C, ${\left[\alpha \right]}_{D}^{20}$ = +145 (c = 0.16, EtOH), *ee* 95%, ^1^H-NMR (600 MHz, CDCl_3_, 30 °C): 2.46–2.52 (m, 1H, CH_2_), 2.70–2.74 (m, 1H, CH_2_), 2.83–2.98 (m, 2H, CH_2_), 3.82 (s, 3H, OCH_3_), 3.96 (s, 3H, OCH_3_), 5.41 (d, *J* = 7.39 Hz, 1H, 3-H), 7.33 (d, *J* = 7.61 Hz, 1H, 4-H) ^13^C-NMR (150 MHz, CDCl_3_, 30 °C): δ *=* 28.8, 30.0, 53.7, 66.0, 81.1, 104.7, 131.1, 165.5, 168.8, 170.6, HRMS calcd. for [M + H^+^] *m*/*z* = 241.0825, found *m*/*z* = 241.0819.

*Methyl (S)-1-methoxy-2,6-dioxo-1,2,6,7,8,8a-hexahydropyrrolo[1,2-a]pyrimidine-8a-carboxylate [(–)-**5**]:* White crystals, (40% yield), m.p. 155–156 °C, ${\left[\alpha \right]}_{D}^{20}$ = −139 (c = 0.16, EtOH), *ee* 99% ^1^H- and ^13^C-NMR is similar to (+)-**5**.

#### 3.2.3. Synthesis of Methanopyrrolo[1,2-*a*]quinazoline Derivatives (+)-**6a**, (–)-**6a**, (+)-**7a**, and (–)-**7a**

A mixture of 2-aminonorbornene hydroxamic acid (+)-**1**, (–)-**1**, (+)-**2**, or (–)-**2** (336 mg, 2 mmol) and levulinic acid (232 mg, 2 mmol) was dissolved in 6 mL EtOH and heated in a 10 mL pressurized reaction vial and the solution was stirred at 100 °C for 1 h at max 300 W microwave irradiation. After that the reaction mixture was evaporated to dryness and the resulting oil was dissolved in dichloromethane and was extracted with 15 mL 20% NaOH solution. The water phase was acidified with 20% HCl solution and extracted with dichloromethane. The organic phase was dried (Na_2_SO_4_) and evaporated. The gained yellow oil was dissolved in MeOH and a solution of diazomethane in Et_2_O was added in a flask protected from light, at room temperature for about 30 min, until no starting material could be observed by TLC. (Diazomethane is a very harmful and hazardous reagent and must be handled with caution! This reaction should be performed in a well-ventilated hood!) The solvent was then removed by evaporation and the residue was dissolved in EtOAc:MeOH = 9:1 (3 mL). The resulting solution was transferred to a silica gel column and eluted with EtOAc:MeOH = 9:1 and the diastereomers were separated. The products were crystallized from *i*Pr_2_O to afford white crystals.

*(3aR,5aS,6R,9S,9aR)-4-methoxy-3a-methyl-2,3,3a,4,5a,6,9a-octahydro-6,9-methanopyrrolo[1,2-a]quinazoline-1,5-dione [(+)-**6a**]:* White crystals (29% yield), m.p. 158–161 °C, ${\left[\alpha \right]}_{D}^{20}$ = +23.1 (*c* = 0.44, EtOH), *ee* 99%, ^1^H-NMR (400 MHz, CDCl_3_, 30 °C): 1.39 (m, 1H, 11-H), 1.53 (s, 3H, CH_3_) 1.57–1.60 (m, 1H, 11-H), 2.12–2.16 (m, 2H, CH_2_), 2.38–2.44 (m, 2H, CH_2_), 3.00–3.04 (m, 1H, 5a-H), 3.45 (m, 1H, 9-H), 3.84 (s, 3H, OCH_3_), 4.03–4.06 (m, 1H, 9a-H), 4.11 (m, 1H, 6-H), 6.09–6.11 (m, 1H, 8-H), 6.18–6.20 (m, 1H, 7-H), ^13^C-NMR (100 MHz, CDCl_3_, 30 °C): δ *=* 23.9, 29.5, 30.8, 45.8, 46.4, 47.0, 48.6, 52.8, 64.8, 80.3, 135.3, 135.6, 170.8, 172.3, HRMS calcd. for [M + H^+^] *m*/*z* = 263.1396, found *m*/*z* = 263.1390.

*(3aS,5aR,6S,9R,9aS)-4-methoxy-3a-methyl-2,3,3a,4,5a,6,9a-octahydro-6,9-methanopyrrolo[1,2-a]quinazoline-1,5-dione [(–)-**6a**]:* White crystals (32% yield), m.p. 157–159 °C, ${\left[\alpha \right]}_{D}^{20}$ = −22.9 (*c* = 0.44, EtOH), *ee* 98%, ^1^H- and ^13^C-NMR is similar to (+)-**6a**.

*(3aR,5aS,6S,9R,9aR)-4-methoxy-3a-methyl-2,3,3a,4,5a,6,9a-octahydro-6,9-methanopyrrolo[1,2-a]quinazoline-1,5-dione [(+)-**7a**]:* White crystals, (28% yield), m.p. 141–142 °C, $\;{\left[\alpha \right]}_{D}^{20}\;$= +62.3 (*c* = 1.12, EtOH), *ee* =87%, ^1^H-NMR (400 MHz, CDCl_3_, 30 °C): 1.61–1.64 (m, 1H, 11-H), 1.68 (m, 3H, CH_3_), 1.80–1.83 (m, 1H, 11-H), 2.17–2.33 (m, 2H, CH_2_), 2.42–2.55 (m, 2H, CH_2_), 2.60–2.63 (m, 1H, 5a-H), 2.81 (m, 1H, 9-H), 3.36 (m, 1H, 6-H), 3.91 (s, 3H, OCH_3_), 4.17–4.19 (m, 1H, 9a-H), 6.19–6.20 (m, 1H, 7-H), 6.39–6.41 (m, 1H, 8-H), ^13^C-NMR (100 MHz, CDCl_3_, 30 °C): δ *=* 22.9, 30.2, 31.3, 44.0, 45.0, 46.3, 48.5, 53.5, 65.3, 81.1, 136.5, 138.7, 166.4, 173.2, HRMS calcd. for [M + H^+^] *m*/*z* = 263.1396, found *m*/*z* = 263.1390.

*(3aS,5aR,6R,9S,9aS)-4-methoxy-3a-methyl-2,3,3a,4,5a,6,9a-octahydro-6,9-methanopyrrolo[1,2-a]quinazoline-1,5-dione [(–)-**7a**]:* White crystals, (31% yield), m.p. 138–141 °C = −72.3 (*c* = 1.18, EtOH), *ee* 91% ^1^H- and ^13^C-NMR is similar to (+)-**7a**.

#### 3.2.4. Synthesis of Pyrrolo[1,2-*a*]pyrimidines (+)-**8** and (–)-**8**

Tetracyclic compound (+)-**6a**, (+)-**7a**, (–)-**6a**, or (–)-**7a** (50 mg, 0.19 mmol) was dissolved in 2 mL DCB in a 10 mL sealed reaction vial. The solution was stirred at 240 °C for 20 min at max 300 W microwave irradiation. Then the solvent was evaporated, and the residue was dissolved in 2 mL EtOAc and purified by column chromatography on silica gel eluted by EtOAc. The product was crystallized from *i*Pr_2_O to produce white crystals.

*(S)-1-methoxy-8a-methyl-1,7,8,8a-tetrahydropyrrolo[1,2-a]pyrimidine-2,6-dione [(+)-**8**]:* White crystals, (55% yield), m.p. 137–140 °C, ${\left[\alpha \right]}_{D}^{20}$ = +158 (*c* = 0.23, EtOH), *ee* 99 %, ^1^H-NMR (400 MHz, CDCl_3_, 30 °C): 1.49 (m, 3H, CH_3_), 2.37–2.46 (m, 2H, CH_2_), 2.51–2.69 (m, 2H, CH_2_), 3.89 (s, 3H, OCH_3_), 5.35 (d, *J* = 7.71 Hz, 1H, 3-H), 7.31 (d, *J* = 7.62 Hz, 1H, 4-H), ^13^C-NMR (100 MHz, CDCl_3_, 30 °C): δ *=* 18.4, 30.0, 32.4, 65.5, 80.8, 104.3, 131.4, 166.3, 171.6, HRMS calcd. for [M + H^+^] *m*/*z* = 197.0926, measured: *m*/*z* = 197.0916.

*(R)-1-methoxy-8a-methyl-1,7,8,8a-tetrahydropyrrolo[1,2-a]pyrimidine-2,6-dione[(–)-**8***]: White crystals, (57% yield), m.p. 139–142 °C; ${\left[\alpha \right]}_{D}^{20}$ = –152 (*c* = 0.23, EtOH), *ee* 99%, ^1^H- and ^13^C-NMR is similar to (+)-**8**.

## 4. Conclusions

In conclusion, new racemic and enantiomeric pyrrolo[1,2-*a*]pyrimidines were synthesized starting from *diendo-* and *diexo*-2-aminonorbornene hydroxamic acids. Their domino ring closure reactions with α-ketoglutaric acid and levulinic acid, and the formation of two diastereomers were observed in each case. After separation by column chromatography, single diastereomers were subjected to a microwave-mediated RDA reaction and gained bicyclic pyrrolo[1,2-*a*]pyrimidines through the loss of cyclopentadiene. When enantiomeric *diexo*- or *diendo*-3-amino-*N*-hydroxybicyclo[2.2.1]hept-5-ene-2-carboxamides were used, the products were enantiomeric heterocycles with *ee* = 95–99%, demonstrating that the starting compounds are excellent chiral sources, and the stereochemical information can be effectively transferable to the newly formed stereogenic centre.

## Supplementary Materials

Supplementary materials are available online.
