# Supplementary material for: Synthesis of Pyrrolo[1,2-a]pyrimidine Enantiomers via Domino Ring-Closure followed by Retro Diels-Alder Protocol
Source: Molecules. 2017 Apr 13;22(4):613. doi: 10.3390/molecules22040613 (PMC6154686; doi:10.3390/molecules22040613)
Supplement: Supplementary file 1 [file molecules-22-00613-s001.pdf]

Supporting information
for
Synthesis of pyrrolo[1,2-*a*]pyrimidine enantiomers via domino ring closure retro Diels-Alder protocol

Beáta Fekete[a], Márta Palkó[a], Matti Haukka[b], Ferenc Fülöp*[a,c]

Address: [a] Institute of Pharmaceutical Chemistry, University of Szeged, H-6720, Szeged, Eötvös utca 6, Hungary; [b] Department of Chemistry, University of Jyväskylä, FIN-40014 Turku, Finland; [c] MTA-SZTE Stereochemistry Research Group, Hungarian Academy of Sciences, H-6720, Szeged, Eötvös utca 6, Hungary

*Corresponding author

Contents

^{1}H-NMR and ^{13}C-NMR spectra..2

HPLC chromatogramms..10

Table for X-ray crystallography data for (±)-**3a**, (±)-**4a** and (±)-**6a** ..12

(3a*S*,5a*S*,6*R*,9*S*,9a*R*)-Methyl 4-methoxy-1,5-dioxo-1,2,3,3a,4,5,5a,6,9a-decahydro-6,9-methanopyrrolo[1,2-*a*]quinazoline-3a-carboxylate [(–)-**3a**)]:

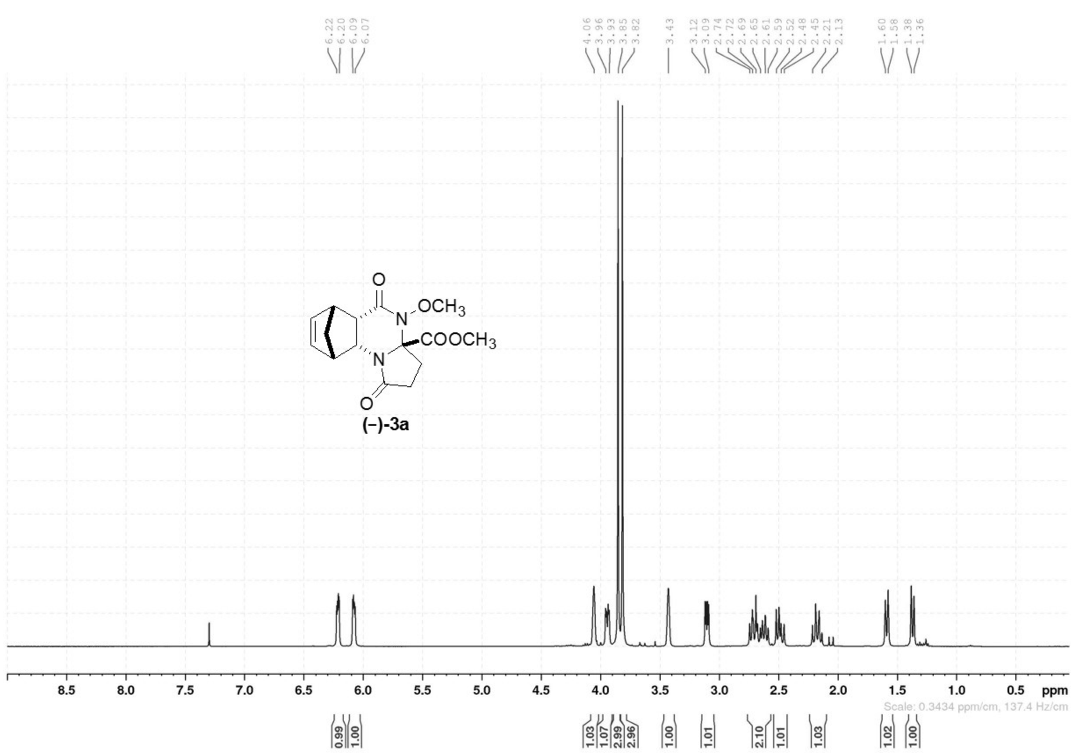

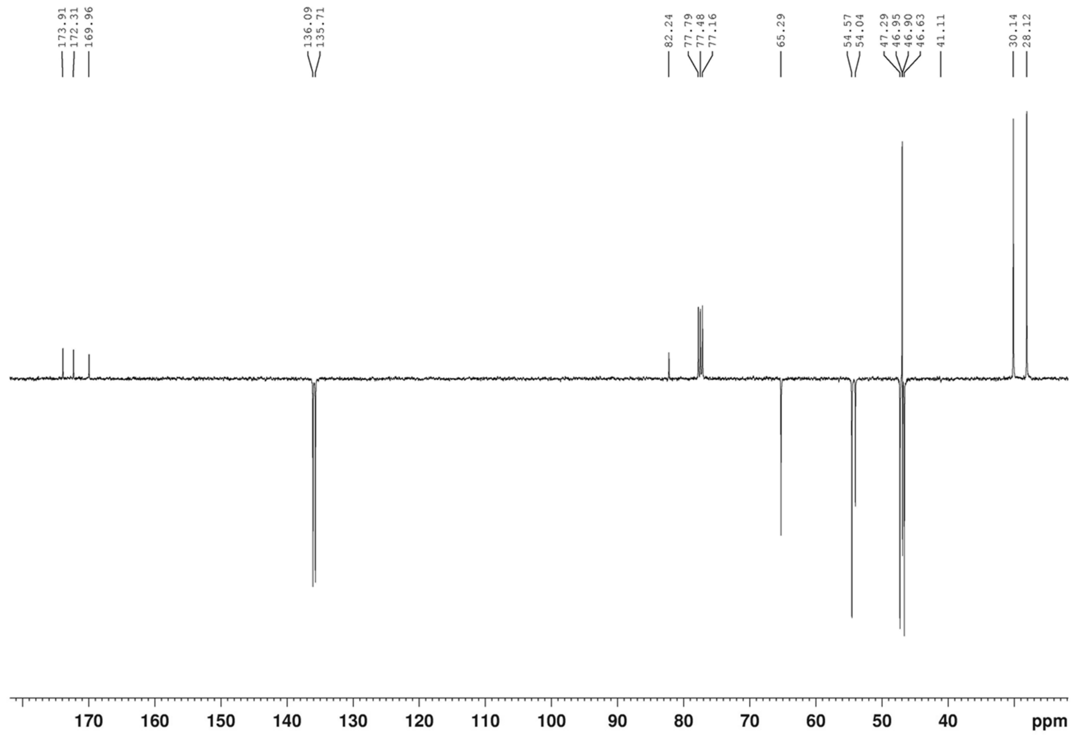

(3a*R*,5a*S*,6*R*,9*S*,9a*R*)-Methyl 4-methoxy-1,5-dioxo-1,2,3,3a,4,5a,6,9a-decahydro-6,9-methanopyrrolo[1,2-*a*]quinazoline-3a-carboxylate [(–)-**3b**)]

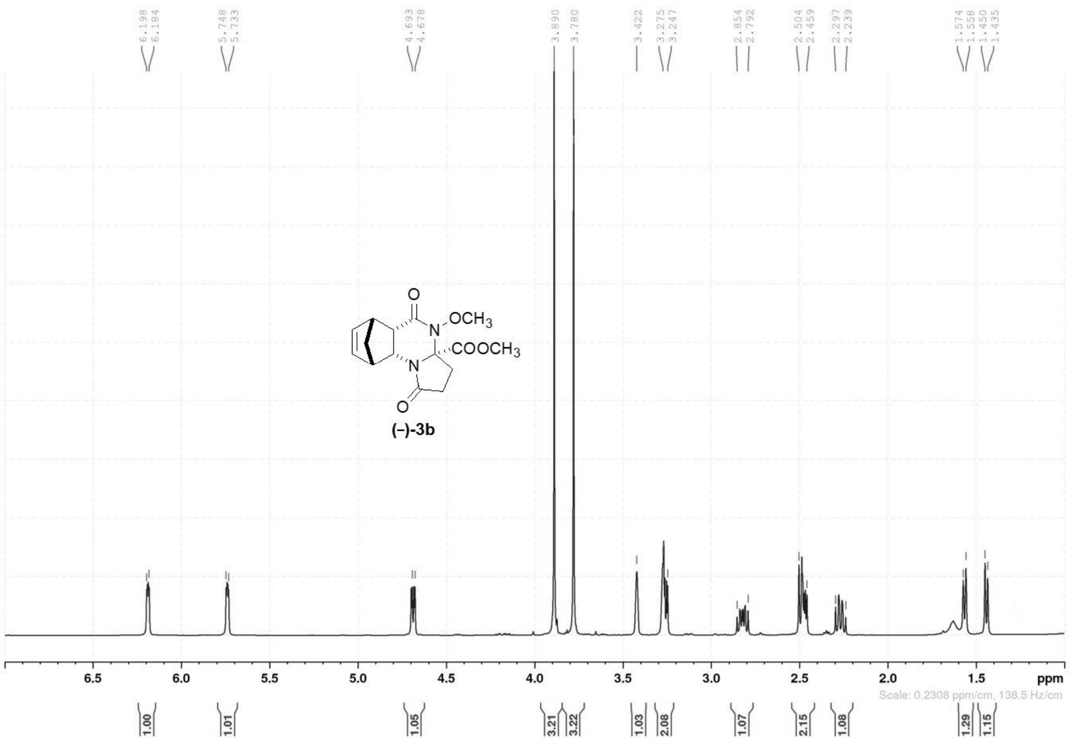

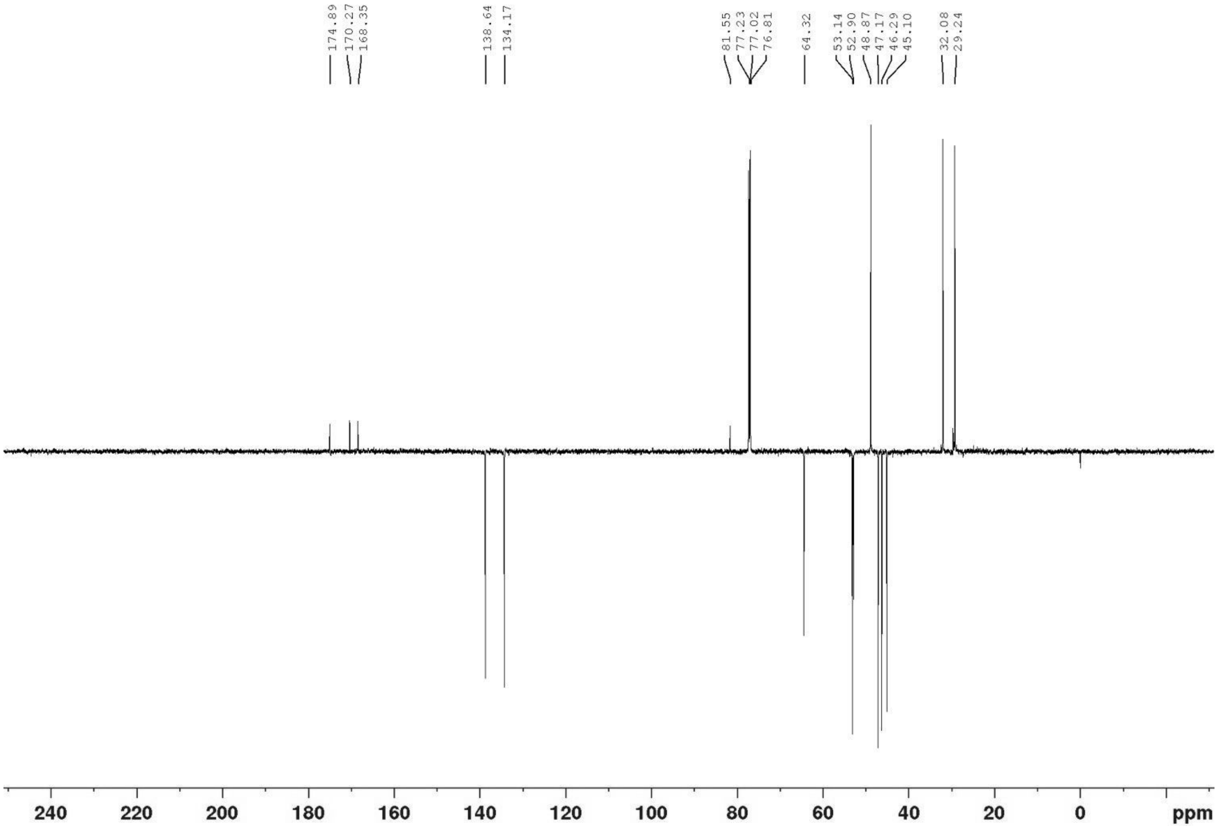

(3a*S*,5a*R*,6*R*,9*S*,9a*S*)-Methyl 4-methoxy-1,5-dioxo-1,2,3,3a,4,5a,6,9a-decahydro-6,9-methanopyrrolo[1,2-*a*]quinazoline-3a-carboxylate [(–)-**4a**)]:

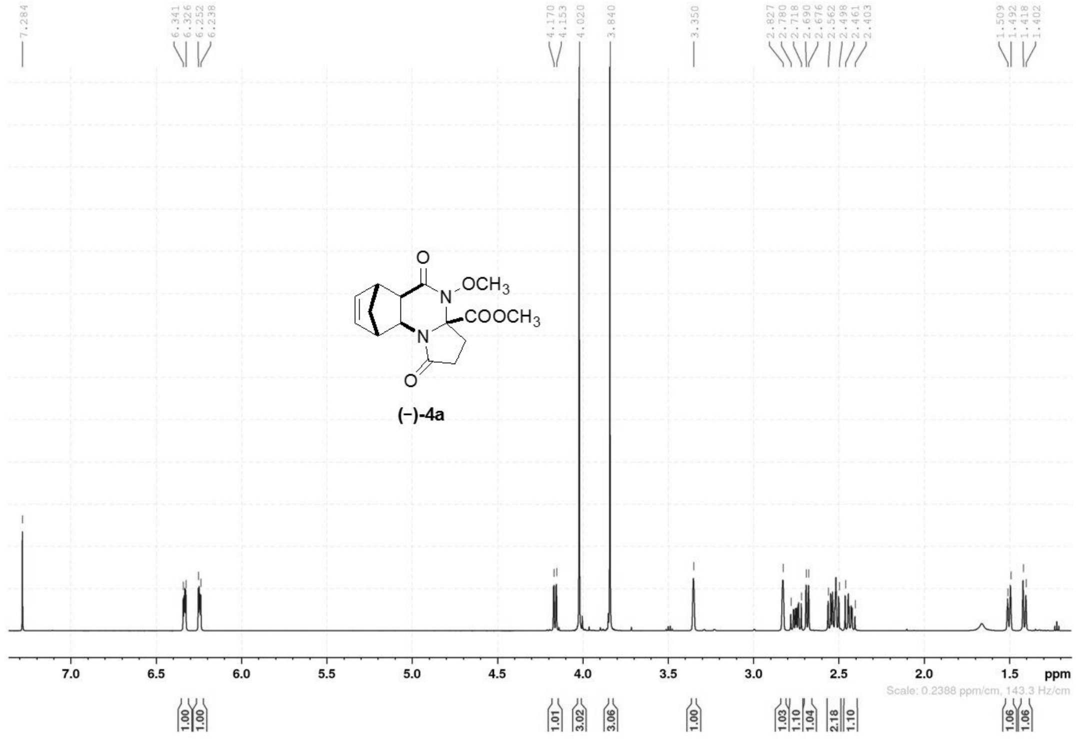

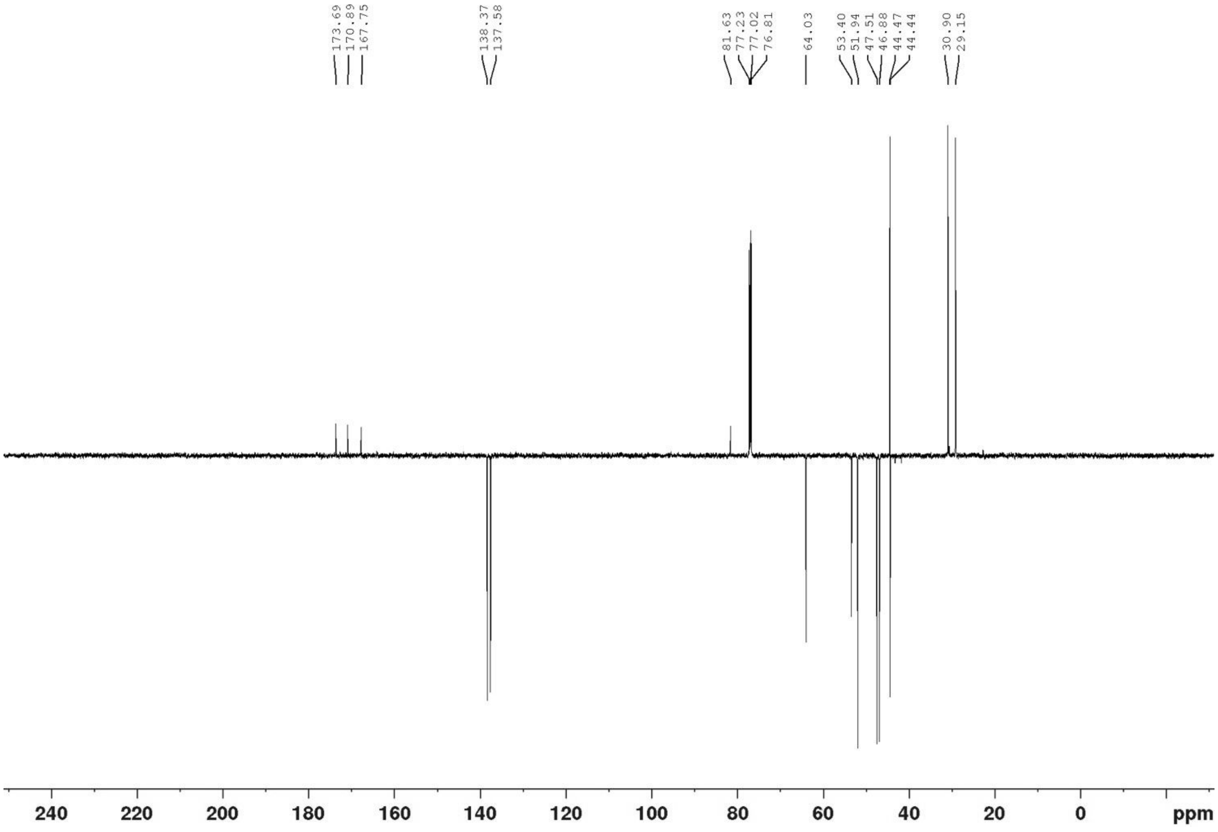

(3a*R*,5a*R*,6*R*,9*S*,9a*S*)-Methyl 4-methoxy-1,5-dioxo-1,2,3,3a,4,5a,6,9a-decahydro-6,9-methanopyrrolo[1,2-*a*]quinazoline-3a-carboxylate [(+)-**4b**]:

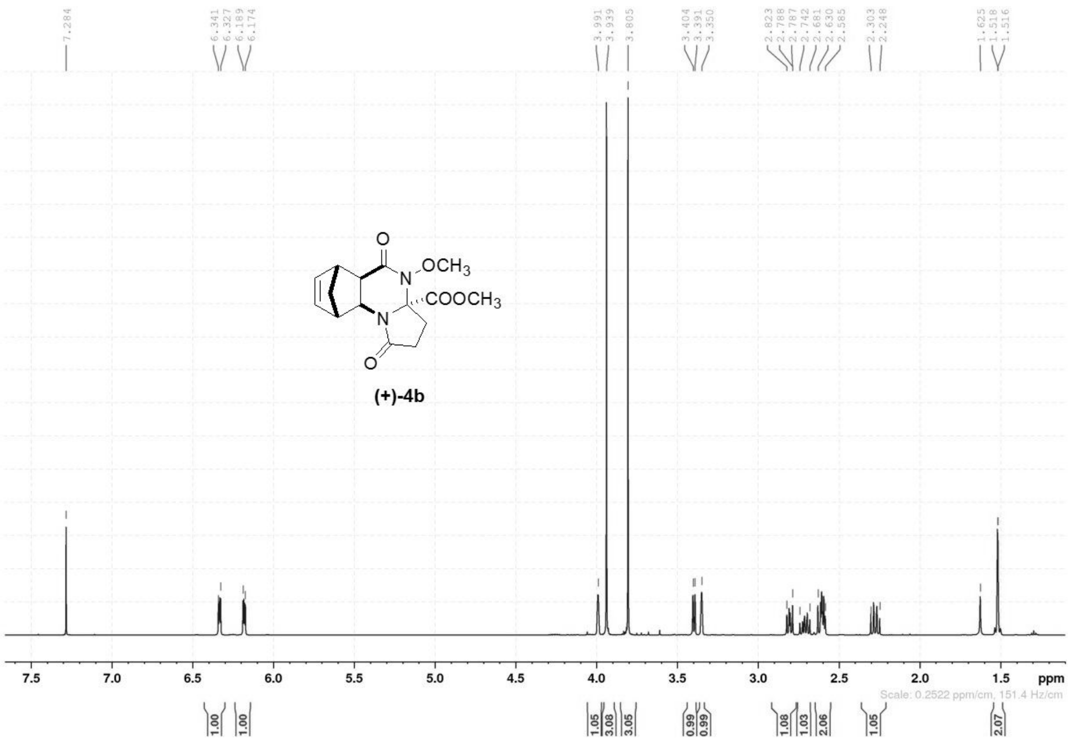

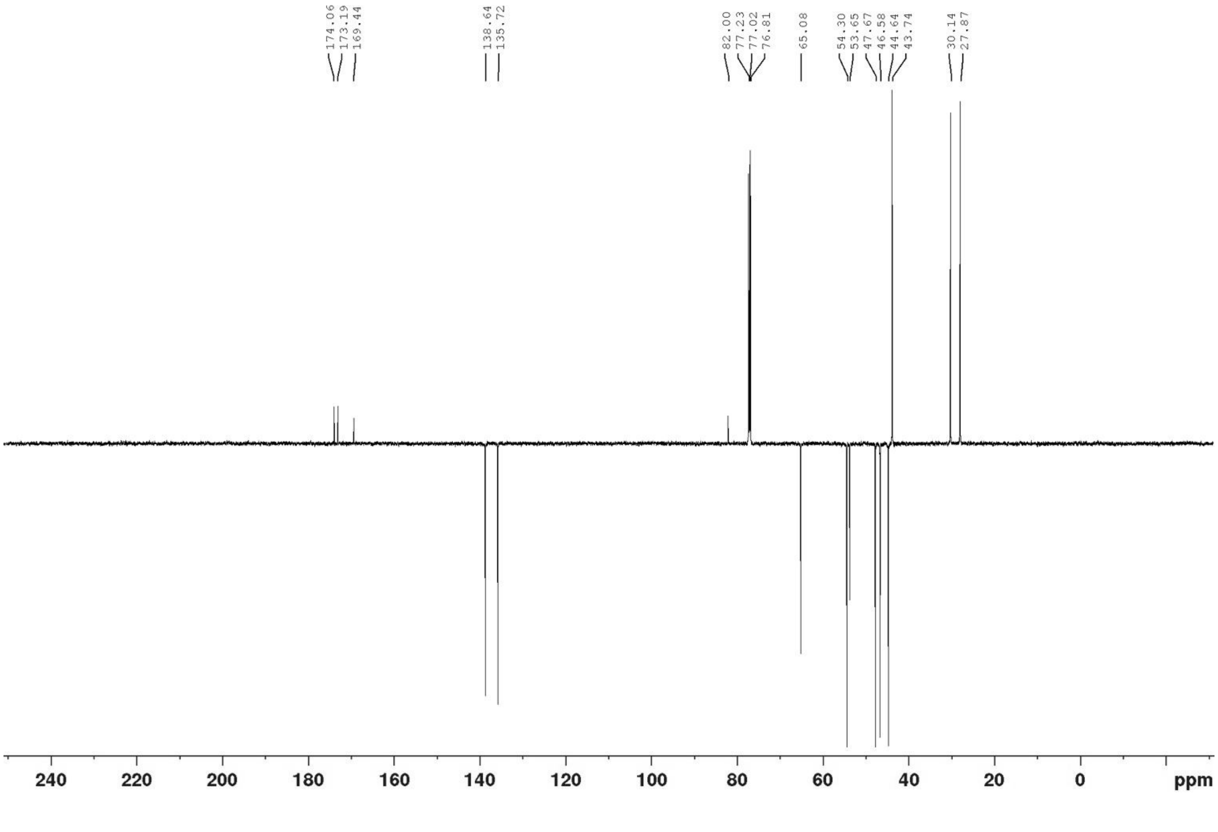

(*R*)-methyl 1-methoxy-2,6-dioxo-1,2,6,7,8,8a-hexahydropyrrolo[1,2-a]pyrimidine-8a-carboxylate [(+)-**5**]:

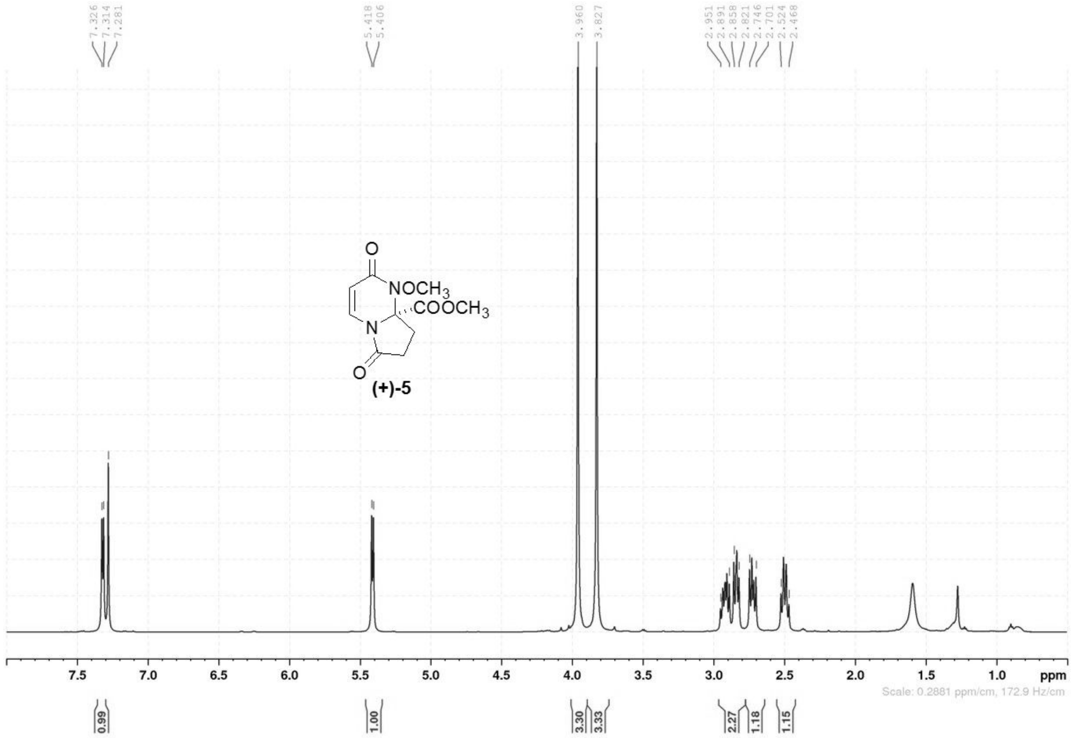

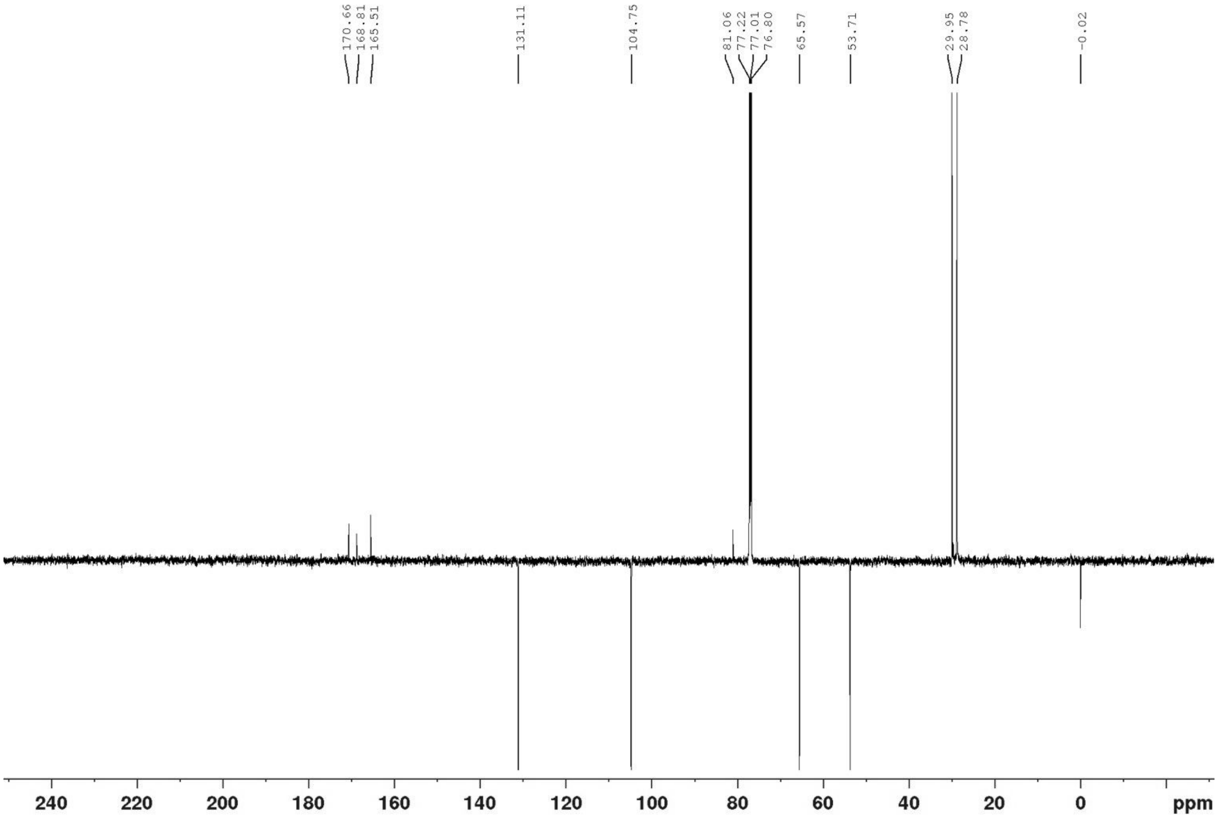

(3a*R*,5a*S*,6*R*,9*S*,9a*R*)-4-methoxy-3a-methyl-2,3,3a,4,5a,6,9a-octahydro-6,9methanopyrrolo[1,2-*a*]quinazoline-1,5-dione [(+)-**6a**]:

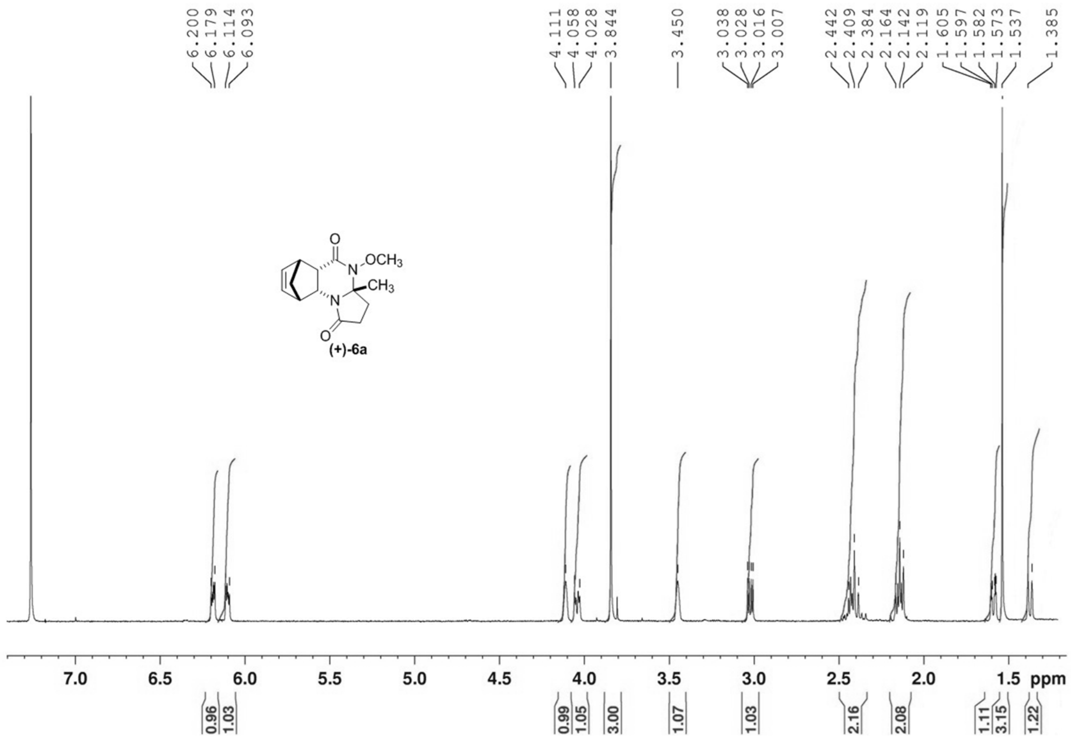

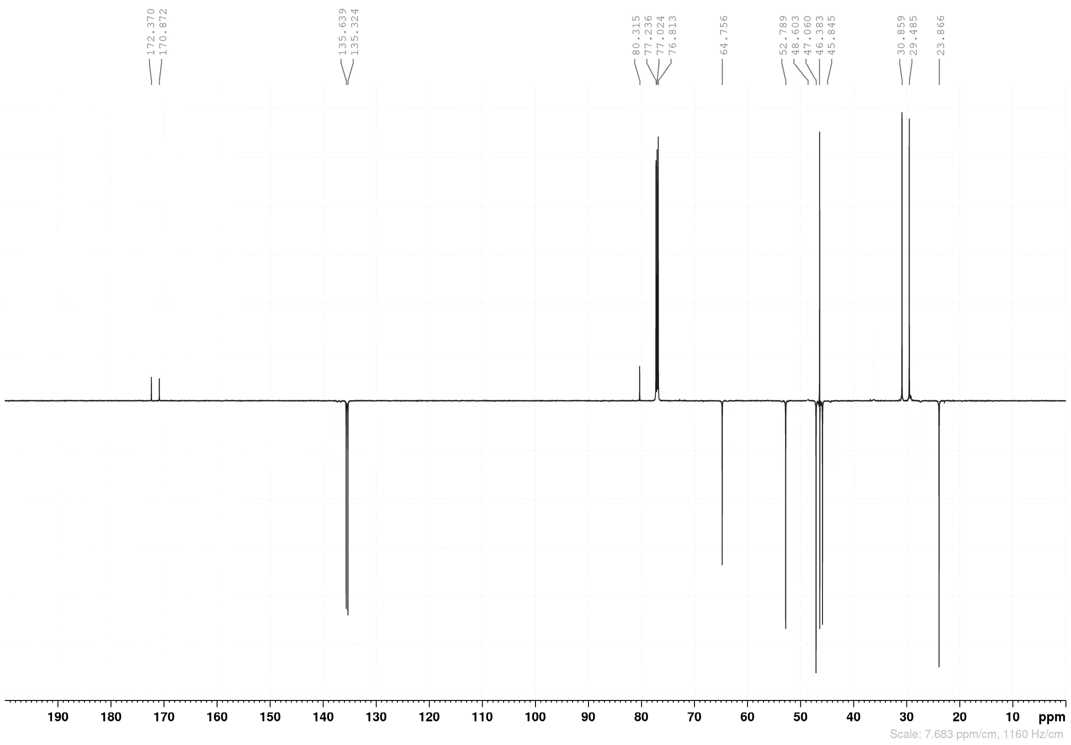

(3a*R*,5a*S*,6*S*,9*R*,9a*R*)-4-methoxy-3a-methyl-2,3,3a,4,5a,6,9a-octahydro-6,9-methanopyrrolo[1,2-*a*]quinazoline-1,5-dione [(+)-**7a**]:

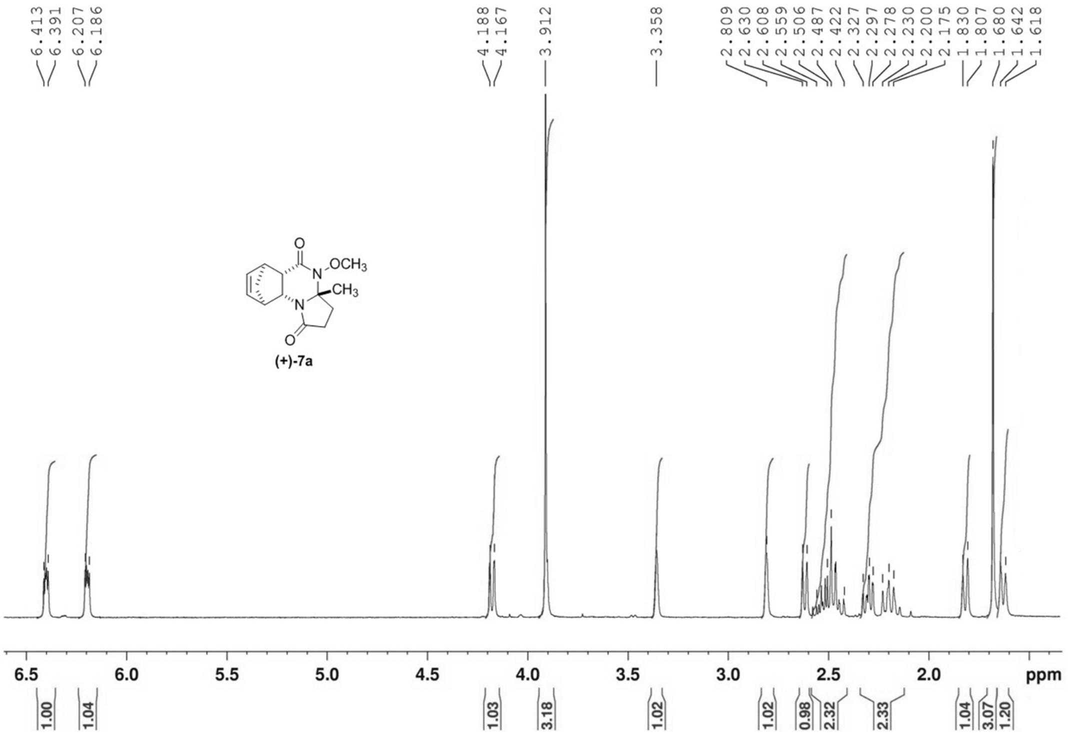

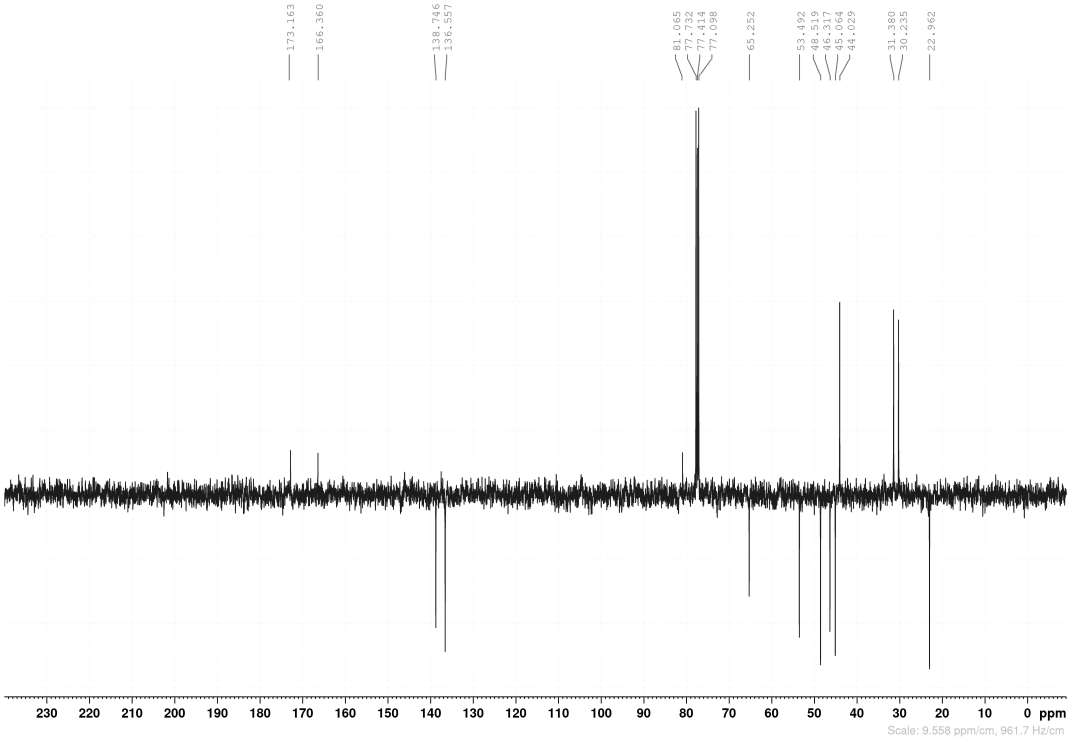

(S)-1-methoxy-8a-methyl-1,7,8,8a-tetrahydropyrrolo[1,2-*a*]pyrimidine-2,6-dione [(+)-**8**]:

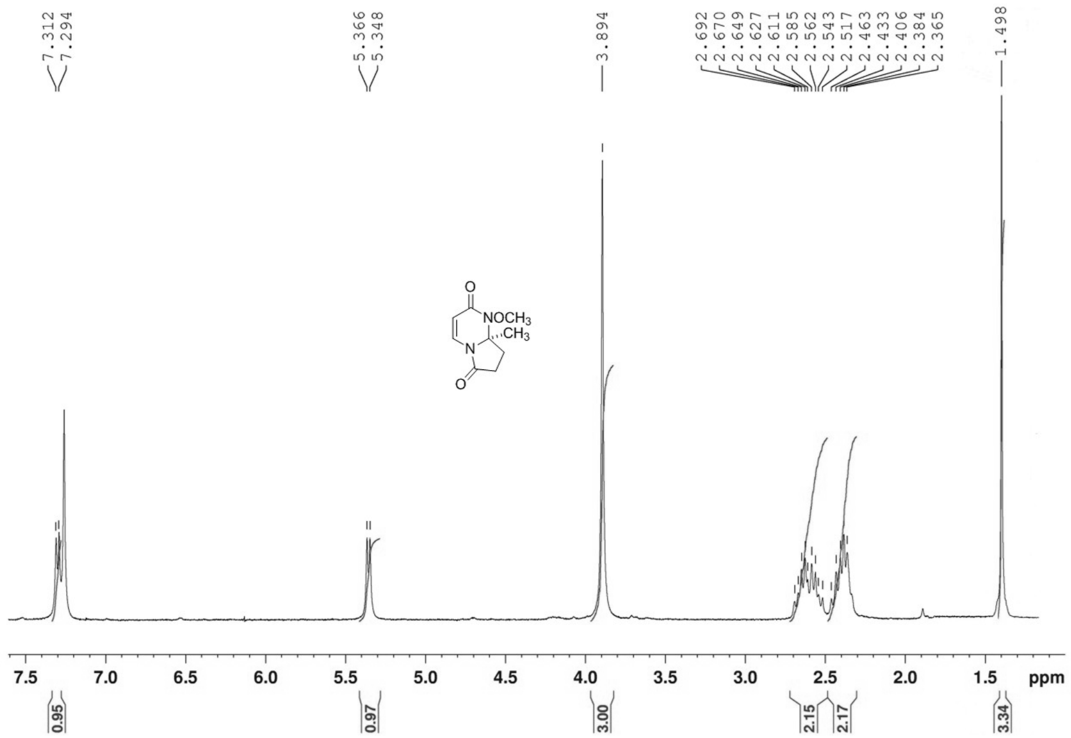

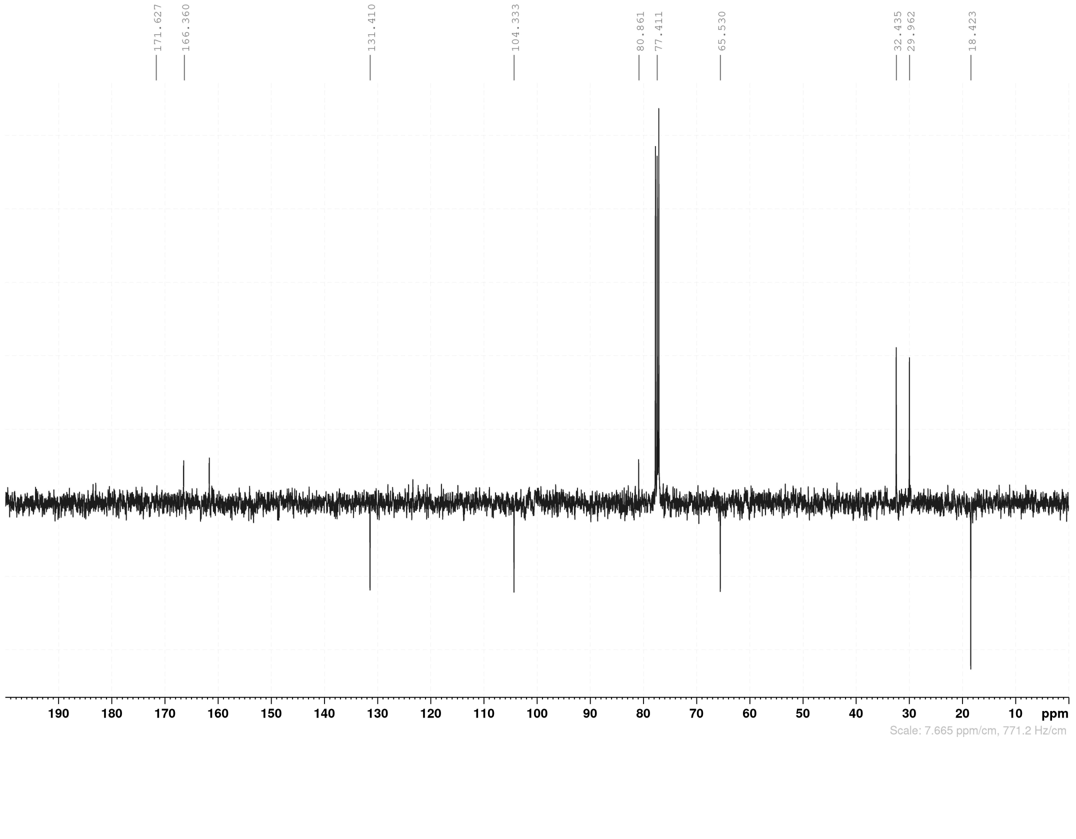

(*RS*)-methyl 1-methoxy-2,6-dioxo-1,2,6,7,8,8a-hexahydropyrrolo[1,2-a]pyrimidine-8a-carboxylate [(±)-**5**]:

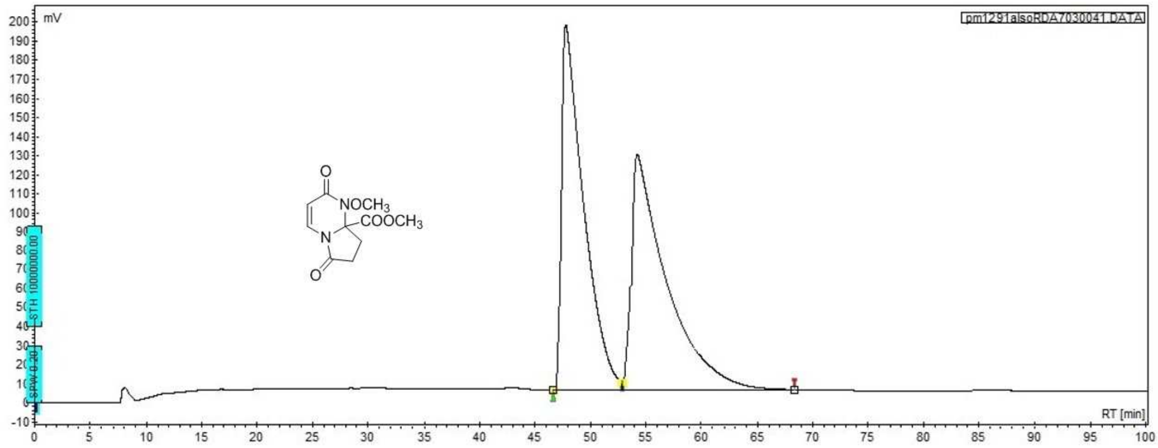

(*R*)-methyl 1-methoxy-2,6-dioxo-1,2,6,7,8,8a-hexahydropyrrolo[1,2-a]pyrimidine-8a-carboxylate [(+)-**5**]:

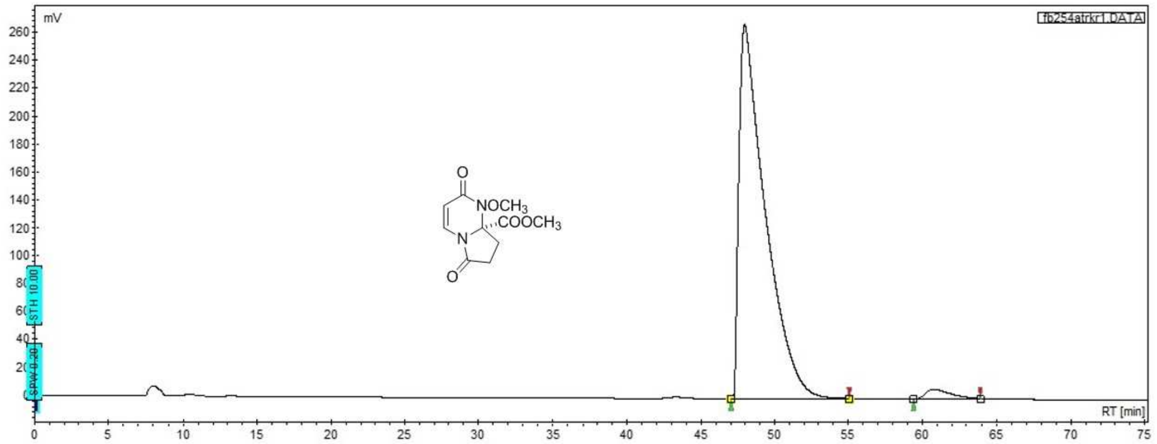

(*S*)-methyl 1-methoxy-2,6-dioxo-1,2,6,7,8,8a-hexahydropyrrolo[1,2-a]pyrimidine-8a-carboxylate [(–)-**5**]:

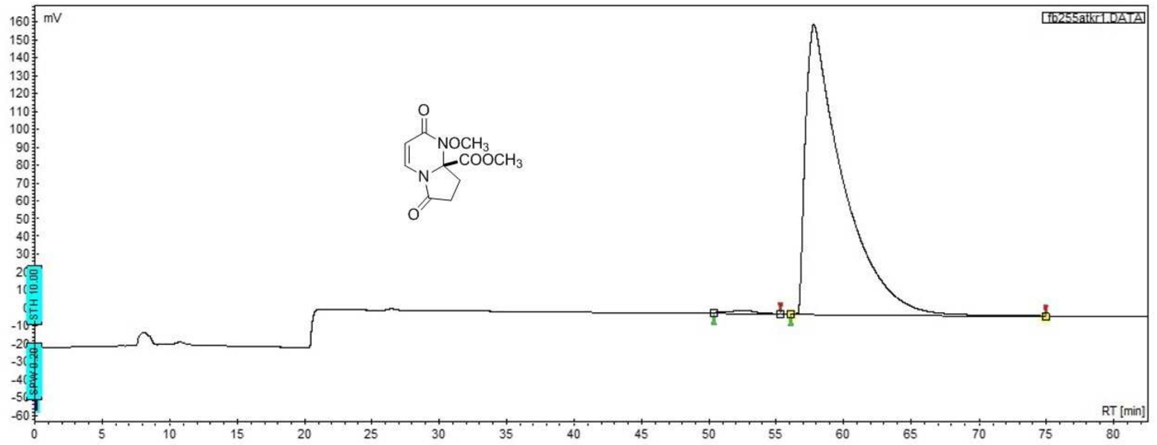

(RS)-1-methoxy-8a-methyl-1,7,8,8a-tetrahydropyrrolo[1,2-*a*]pyrimidine-2,6-dione [(±)-**8**]:

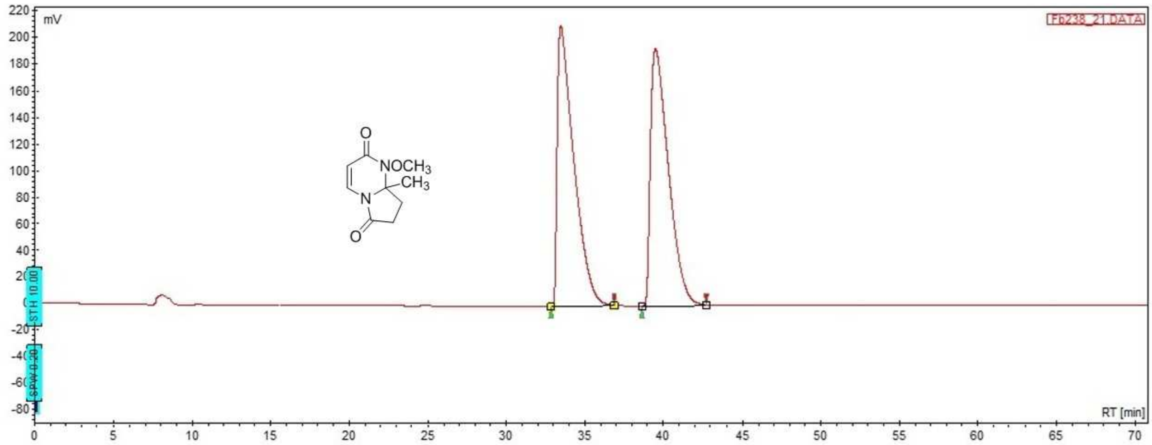

(S)-1-methoxy-8a-methyl-1,7,8,8a-tetrahydropyrrolo[1,2-*a*]pyrimidine-2,6-dione [(+)-**8**]:

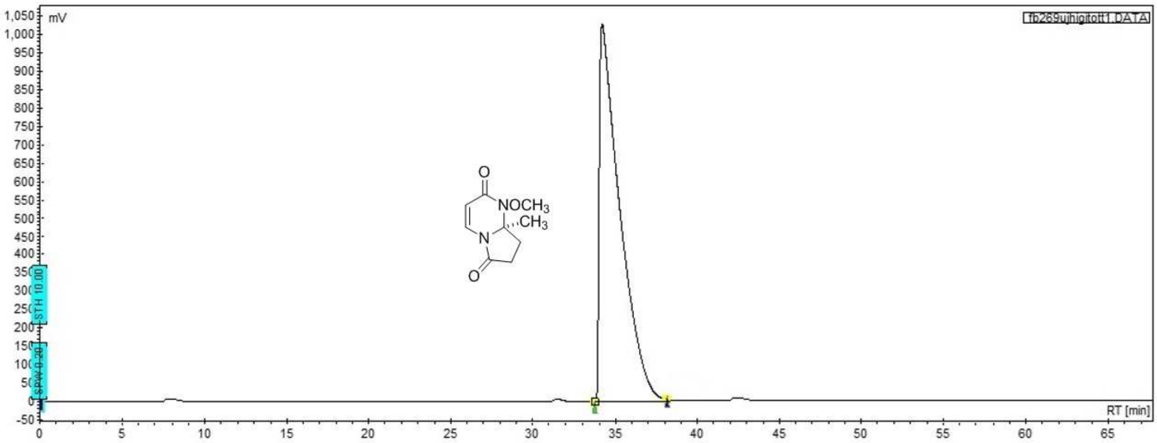

(R)-1-methoxy-8a-methyl-1,7,8,8a-tetrahydropyrrolo[1,2-*a*]pyrimidine-2,6-dione [(–)-**8**]:

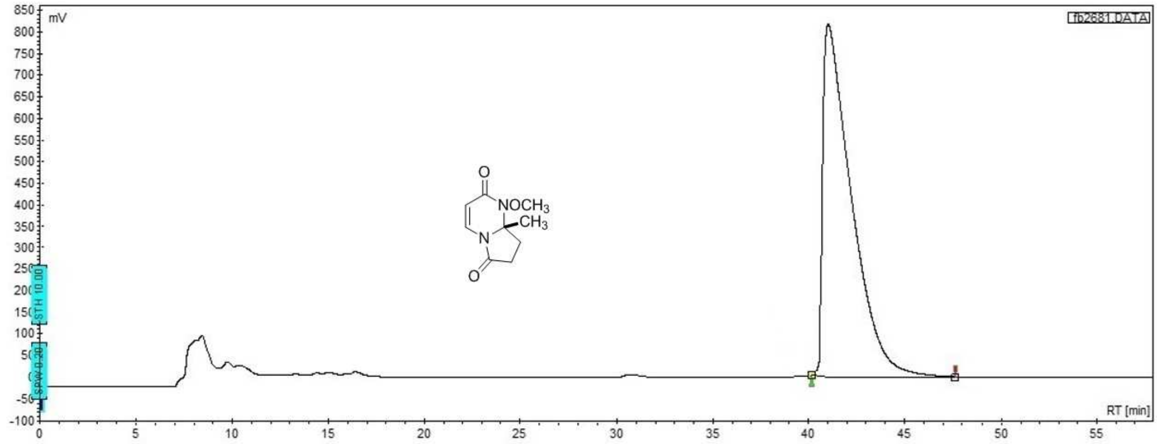

Table for X-ray crystallography data for (±)-**3a**, (±)-**4b** and (±)-**6a**

	(±)-**3a**	(±)-**4b**	(±)-**6a**
Empirical formula	$C_{15}H_{18}N_2O_5$	$C_{15}H_{18}N_2O_5$	$C_{14}H_{18}N_2O_3$
Formula weight	306.31	306.31	262.30
Temperature	120(2) K	120(2) K	120(2) K
Wavelength	1.54184 Å	1.54184 Å	0.71073 Å
Crystal system	Triclinic	Monoclinic	Monoclinic
Space group	$P\bar{1}$	$P2_1/c$	$P2_1/n$
Unit cell dimensions	a = 8.3315(3) Å<br>α= 81.778(3)	a = 16.7015(3) Å<br>α= 90	a = 9.4473(3) Å<br>α= 90º
	b = 8.7788(3) Å<br>β= 82.075(3)	b = 9.4689(3) Å<br>β= 98.6847(17)°	b = 10.0314(2) Å<br>β= 105.075(3)º
	c = 9.8739(3) Å<br>γ = 83.141(2)	c = 18.1845(4) Å<br>γ = 90	c = 13.7952(4) Å<br>γ = 90º
Volume	704.26(4) $Å^3$	2842.82(11) $Å^3$	1262.36(6) $Å^3$
Z	2	8	4
Density (calculated)	1.444 Mg/m^3	1.431 Mg/m^3	1.380 Mg/m^3
Absorption coefficient	0.916 mm^{-1}	0.908 mm^{-1}	0.098 mm^{-1}
F(000)	324	1296	560
Crystal size	0.191 x 0.152 x 0.081 mm^3	0.702 x 0.498 x 0.299 mm^3	0.418 x 0.391 x 0.243 mm^3
Theta range for data collection	4.559 to 76.864°.	4.920 to 76.944°.	3.110 to 29.575º.
Index ranges	-10<=h<=10, -11<=k<=11, -12<=l<=12	-21<=h<=21, -11<=k<=11, -22<=l<=22	-12<=h<=12, -13<=k<=13, -19<=l<=18
Reflections collected	15453	44312	20374
Independent reflections	2963 [R(int) = 0.0573]	5930 [R(int) = 0.0496]	3501 [R(int) = 0.0432]
Completeness to theta	67.684°<br>99.7 %	67.684°<br>98.9 %	26.000º<br>99.8 %

Refinement method	Full-matrix least-squares on F^2	Full-matrix least-squares on F^2	Full-matrix least-squares on F^2
Data / restraints / parameters	2963 / 0 / 201	5930 / 0 / 401	3501 / 0 / 174
Goodness-of-fit on F^2	1.070	1.072	1.046
Final R indices [I>2sigma(I)]	R1 = 0.0432, wR2 = 0.1170	R1 = 0.0485, wR2 = 0.1283	R1 = 0.0434, wR2 = 0.1110
R indices (all data)	R1 = 0.0439, wR2 = 0.1177	R1 = 0.0488, wR2 = 0.1286	R1 = 0.0517, wR2 = 0.1181
Extinction coefficient	n/a	n/a	n/a
Largest diff. peak and hole	0.350 and -0.280 e.Å^{-3}	0.335 and -0.275 e.Å^{-3}	0.286 and -0.225 e.Å^{-3}
